# FATAL SEPTIC SHOCK DUE TO WEEKSELLA VIROSA IN 69-YEAR-OLD MALE

**DOI:** 10.51894/001c.123109

**Published:** 2024-08-30

**Authors:** Baral Dilip

**Affiliations:** 1 Transitional Program Detroit Medical Centre Sinai-Grace Hospital

## 89

### INTRODUCTION

*Weeksella virosa*, an aerobic Gram-negative rod, is an uncommon but potentially fatal cause of sepsis and septic shock. Existing literature on this pathogen is limited, with most reported cases linking it to severe outcomes in immunocompromised individuals with multiple comorbidities. In this case study, we present a 69-year-old male with a history of chronic Foley catheterization due to urinary retention, hypertension, hyperlipidemia, and benign prostatic hyperplasia, who was admitted to the emergency department in an altered mental state.

### CASE DESCRIPTION

Upon arrival, the patient exhibited signs of septic shock, with a significantly distended abdomen and pus around the urethral meatus. The Foley catheter was replaced, draining substantial amounts of purulent material. Initial vital signs indicated hypotension (BP 70/50 mmHg) and tachycardia (HR 114/min), accompanied by laboratory findings revealing a white blood cell count of 36.8 x10^9^/L, hyperkalemia (potassium 8.3 mmol/L), elevated BUN (240 mg/dL), and creatinine (13.57 mg/dL). Blood and urine cultures were obtained, revealing *Weeksella virosa* as the causative agent. Despite aggressive interventions, including fluid resuscitation, antibiotic therapy (initially with cefepime), and continuous renal replacement therapy (CRRT) for metabolic derangements, the patient’s condition rapidly deteriorated. Maximal vasopressor support failed to stabilize blood pressure, culminating in cardiac arrest on the fourth day of admission. Postmortem blood cultures confirmed *Weeksella virosa* susceptibility to various antibiotics.

### CONCLUSION

This case underscores the potential lethality of *Weeksella virosa*-induced septic shock, especially in patients with underlying health complexities. The swift recognition of septic shock, coupled with prompt initiation of intravenous fluids and empiric antibiotics, is crucial. Blood cultures play a pivotal role in refining antibiotic selection. While *Weeksella virosa* infections remain rare, healthcare providers should be vigilant in considering this pathogen, particularly in immunocompromised individuals, to enhance timely and targeted therapeutic interventions. Further research is warranted to elucidate the optimal management strategies for this rare but potentially devastating infection.

